# Tandem resection of multiple spinal schwannomas

**DOI:** 10.3171/2023.7.FOCVID2393

**Published:** 2023-10-01

**Authors:** Joseph S. Bell, Ulrich Batzdorf, Langston T. Holly

**Affiliations:** Department of Neurosurgery, University of California, Los Angeles, California

**Keywords:** intradural spinal tumor, schwannomatosis, laminoplasty

## Abstract

This video depicts the resection of three separate intradural extramedullary spinal tumors performed under the same anesthetic. Neuromonitoring was used to identify motor nerve roots, and laminoplasty was performed at the thoracolumbar junction to preserve alignment and minimize the risk of postoperative CSF leak.

## Transcript

This video depicts the resection of three separate spinal tumors performed under the same anesthetic.

### 0:26 Patient Presentation.

This patient is a previously healthy 35-year-old man who presented for evaluation of chronic low-back and right leg pain. In the course of his workup, he underwent MRI of the thoracic and lumbar spine, which demonstrated three well-circumscribed, T2-hyperintense, avidly contrast-enhancing intradural extramedullary tumors. The tumors were seen at the level of the conus medullaris at T12/L1, at the level of the L3 body, and at S1/S2. Further imaging revealed a smaller tumor at the left C4–5 neuroforamen, but no evidence of intracranial lesions including in the internal auditory canals.^1^ The patient desired surgical resection of his lumbar tumors and preferred they be treated in a single stage. The patient’s young age, good health status, and home support system were considered to be factors that would allow him to undergo and recover from the extended surgical time required for single-stage resection, so he was offered resection of all three tumors in the same setting.

### 1:21 Patient Positioning and Exposure.

The patient was positioned prone on a Jackson table with his arms extended above his head. SSEP, MEP, and EMG were performed.^2^ We began at the T12/L1 level, which was localized using AP and lateral fluoroscopy. A midline incision was made and monopolar cautery was used to perform a subperiosteal dissection over the lamina of T12 and L1. The interspinous ligament was resected at each end of this segment, and then the burr was used to drill laminar troughs and this segment was removed en bloc to allow for laminoplasty.^3^ Laminoplasty was performed for this segment because of the increased risk of deformity with laminectomy at the junctional level.

### 1:58 Resection of the T12/L1 Tumor.

The dura was opened in the midline and retracted with suture, followed by the arachnoid. The tumor capsule was coagulated and then opened sharply, and the tumor was debulked with an ultrasonic aspirator. Adherent nerve roots were removed from the tumor using sharp dissection. One nerve root was incorporated into the tumor itself and was coagulated and divided.^4^ The tumor was then able to be elevated out of the spinal canal. Meticulous hemostasis was achieved, and then the dura was closed in a watertight fashion using 4-0 Nurolon suture, reinforced by DuraGen and fibrin glue. The lamina of T12 and L1 were then refixed in place using laminoplasty plates and a layered closure was performed.

### 3:32 Resection of the S1/S2 Tumor.

Attention was then turned to the caudal tumor, which was eccentric to the right at the level of S1 and S2. After localizing with lateral fluoroscopy, a second midline incision was opened and a laminectomy of S1 and S2 were performed. The tumor was predominantly extradural and displaced the thecal sac to the patient’s left. The tumor capsule was coagulated and opened, and the tumor debulked. After debulking the traversing nerve root was visible ventral to the tumor. This was preserved, and the tumor was removed using sharp dissection. The rostral end of the tumor was followed and found to enter the dura. The dura was opened and microdissectors used to extract the intradural component. The dura was then closed using AnastoClips, and the closure reinforced with DuraGen and fibrin glue. The wound was again closed in layers.

### 4:56 Resection of the L3 Tumor.

Finally, attention was turned to the tumor at L3. A third incision was created and an L3 laminectomy was performed. The dura was again opened in the midline and retracted, exposing the tumor nestled among the lumbar nerve roots. Adherent nerve roots were sharply dissected. One adherent root splayed across the tumor could not be dissected free. This nerve was stimulated at 2 mA and did not produce any detectable EMG signal, and was coagulated and divided to release the tumor.^5,6^ The dura was again closed in a watertight fashion, and the wound closed in layers.

### 6:19 Postoperative Course.

The patient had an uneventful postoperative course. He was kept on flat bedrest for 72 hours postoperatively to reduce the risk of cerebrospinal fluid leak and was discharged from the hospital on postoperative day 4. Tumor pathology of all tumors was consistent with schwannoma, and postoperative MRI did not reveal any residual enhancing tumor. Genetic testing confirmed the presence of a loss-of-function mutation in the NF2 gene, confirming the diagnosis of NF2-related schwannomatosis. The patient was seen on follow-up with postoperative x-ray demonstrating preservation of his spinal alignment. His preoperative symptoms have completely resolved.

## Disclosures

The authors report no conflict of interest concerning the materials or methods used in this study or the findings specified in this publication.

## Author Contributions

Primary surgeon: Holly. Assistant surgeon: Bell, Batzdorf. Editing and drafting the video and abstract: Holly, Bell. Critically revising the work: Holly, Bell. Reviewed submitted version of the work: Holly, Bell. Approved the final version of the work on behalf of all authors: Holly. Supervision: Holly.

## Supplemental Information

### Patient Informed Consent

The necessary patient informed consent was obtained in this study.
